# Field Studies Evaluating Bait Acceptance and Handling by Free-Roaming Dogs in Thailand

**DOI:** 10.3390/vetsci5020047

**Published:** 2018-05-04

**Authors:** Suwicha Kasemsuwan, Karoon Chanachai, Tanu Pinyopummintr, Kansuda Leelalapongsathon, Kitipat Sujit, Ad Vos

**Affiliations:** 1 Faculty of Veterinary Medicine, Kasetsart University, Kamphaeng Saen 73140, Thailand; fvetswk@ku.ac.th (S.K.); fvettnp1@yahoo.com (T.P.); fvetkul@ku.ac.th (K.L.); 2 Department of Livestock Development, Ministry of Agriculture, Ratchathewi, Bangok 10400, Thailand; kchanachai@hotmail.com (K.C.); kitipat_sujit@hotmail.com (K.S.); 3 IDT Biologika GmbH, Am Pharmapark, 06861 Dessau-Rosslau, Germany

**Keywords:** rabies, bait, dog, oral vaccination

## Abstract

(1) Background: As part of the ongoing endeavor to eliminate dog-mediated human rabies in Thailand, renewed interest has been shown in oral vaccination of dogs as a supplementary tool to increase vaccination coverage of the dog population. (2) Methods: Three different bait types were tested using a hand-out model on the campus of the Kasetsart University and the surrounding temples in Thailand during September 2017, consisting of two industrial manufactured baits (fish meal and egg-flavored) and one bait made from local material (boiled pig intestine placed in collagen casing). A PVC-capsule containing dyed water was inserted in the bait. (3) Results: The fishmeal bait was significantly less often accepted and consumed (50.29%) than the other two baits (intestine bait—79.19%; egg bait—78.77%). Delivery and release of the dyed water in the oral cavity was highest in the egg-flavored bait (84.50%), followed by the intestine bait (76.61%) and fishmeal (54.85%) baits. Bait acceptance was influenced by sex, age, and body size of the dog. Also, the origin of the dogs had a significant effect: temple dogs accepted the baits more often than street dogs. (4) Conclusion: A significant portion of the free-roaming dog population in this study can be vaccinated by offering vaccine baits.

## 1. Introduction

The rabies situation in Thailand, with the dog as major reservoir species, has improved considerably in the last decades. In 1980 and 2015, 370 and 5 human cases were reported, respectively [1,2]. In addition, the number of animal cases has shown a drastic decrease [1]. Two factors have contributed predominantly to this positive development: increased numbers of people receiving PEP and enhanced efforts to vaccinate dogs [2,3,4]. For example, overall dog vaccination coverage has increased from 28% (1993) to 71% (2000) [5]. However, the aim of the Government to reach 80% dog vaccination coverage and eliminate dog-mediated rabies in Thailand has not yet been achieved in all areas of the country. For example, a serological survey among free-roaming dogs in different settings of the capital Bangkok showed marked differences between the central and outskirt areas of the city, with 86% and 49% of the dogs testing seropositive, respectively [6].

Most dogs in Thailand are free-roaming and many are not owned [7]. A special group of these free-roaming dogs are so-called temple dogs. Large groups of dogs can be found around the Buddhist temples in Thailand. Some are owned or taken care of by the monks, and also by people living near the temples who look after the animals. Sometimes, these dogs are vaccinated against rabies or even sterilized, but often no-one claims direct responsibility for these dogs [8]. Most human rabies cases are a direct result of rabid free-roaming dog bites [8], as the vaccination coverage of this subpopulation is low due to inadequate accessibility [9]. Hence, alternative strategies to improve the vaccination coverage of inaccessible dogs must be considered for parenteral vaccination. Oral vaccination campaigns targeted at different wildlife species have been shown to be highly effective [10] and, for example, have led to the almost complete elimination of fox-mediated rabies in the European Union [11]. Hereby, a vaccine-loaded capsule is incorporated in a bait attractive to the target species and distributed in the environment. Upon consumption, the animal punctures the capsule with its teeth and subsequently the vaccine is released in the oral cavity where it is predominantly taken up by the palatine tonsils, followed by the induction of a protective immune response [12]. As a result of the successes obtained with wildlife, oral vaccination against rabies (ORV) has been suggested for domestic dogs inaccessible for parenteral vaccination [13]. As mentioned, a large percentage of dogs in Thailand are free-roaming and inaccessible for parenteral vaccination. Hence, the veterinary authorities have investigated the possibility of ORV in dogs [14]. The present study represents renewed efforts to assess the potential of ORV of dogs in Thailand, especially considering the recent increase in reported rabies cases. As one of the essential components of ORV is a bait well accepted by the target population, the objective of this study was to evaluate bait handling and the acceptance of baits by local free-roaming dogs.

## 2. Materials and Methods 

**Figure 1:**
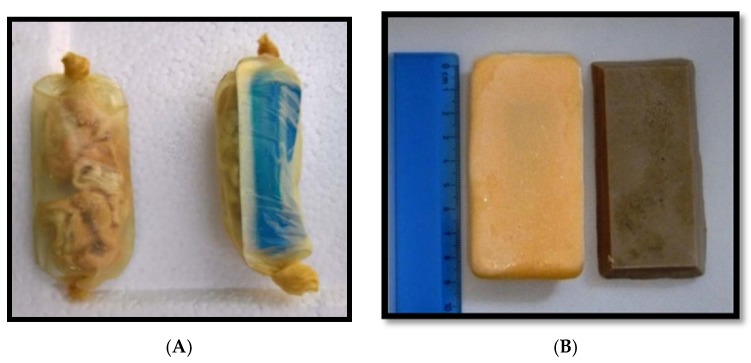
The intestine bait with (**A**) and experimental egg-flavored bait (yellow) and fishmeal bait (brown) (**B**). All bait types contained a vaccine capsule filled with dyed water as can be seen in the intestine bait.

Three different baits were tested: intestine, fishmeal, and egg-flavored baits (Table 1 and Figure 1). The two industrial manufactured baits, the fishmeal and egg-flavored baits, were identical to the baits used in a previous study [15]. The intestine bait was prepared manually and was slightly adapted due to problems encountered during its initial preparation. This bait consisted of a collagen casing (Nippi Casing #300, Nippi Incorp., Tokyo, Japan) filled with pieces of boiled pork intestine and the capsule. All PVC-capsules were sealed with aluminum foil (6.5 × 3.0 × 0.7 cm) and contained a food colorant (Patent Blue V, Thermo Fischer Scientific, Geel, Belgium) dissolved in water (3.5 mL) and no active ingredients. 

Five teams consisting of 3–5 persons visited their allocated sections of the study area and systematically searched for the pre-identified dogs between 06:00 and 19:00 during 18–20 September 2017. Prior to the actual bait study, the dog population had been surveyed and the locations where the dogs could be encountered were identified, including the number of dogs present. In case the care-takers of the dogs were present, oral consent to include the dogs in the trial was obtained and, optionally, the care-takers were given a leaflet that contained additional information and a phone number and e-mail address to report any potential adverse reactions. The type of bait offered was randomly pre-determined and the type of bait offered to the dog was concealed to the person offering the bait until the actual attempt. A form was filled out during every baiting attempt to collect data on bait acceptance and handling, including handling time (time spent by the dog manipulating the bait) and if the vaccine capsule was perforated, discarded, or swallowed. It was also recorded if the discarded capsule was recollected or not. A final assessment was made by the observer if the dog would most likely have been successfully vaccinated based on the release of the dyed-water in the oral cavity (“efficacy”). Additional information recorded for each dog included: ownership, level of supervision, sex, age, and body size. The following subpopulations of dogs (ownership) were tested: owned dog (with identifiable owner), temple dog (dog living within the premises of a temple), street dog (dog with no identifiable owner but living in close proximity of humans—community dogs) and feral dog (unowned dogs avoiding human proximity). If the dog discarded the vaccine blister or if the dog did not accept the bait, the bait was recollected by the observer. A multivariate analysis of a previous data set from the Navajo Nation, USA, based on a similar protocol resulted in an extremely poor fitted model predominantly due to the fact that most factors examined were qualitative instead of quantitative [15]. Hence, univariate data analysis using the statistical software package GraphPad Prism 6 was applied for this study. 

### Study Area

The study was carried out on the campus (3000 acres) of the Kasetsart University and the nearby temples in Kamphaeng Saen, Nakhon Pathom, and the lower central region of Thailand, approximately 80 km northwest from the capital Bangkok. 

## 3. Results

**Figure 2:**
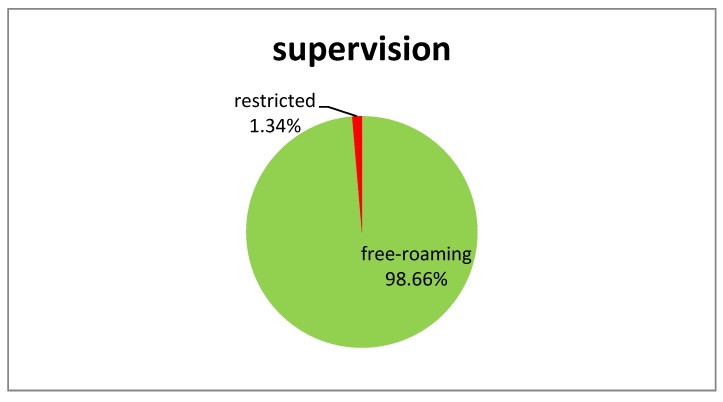

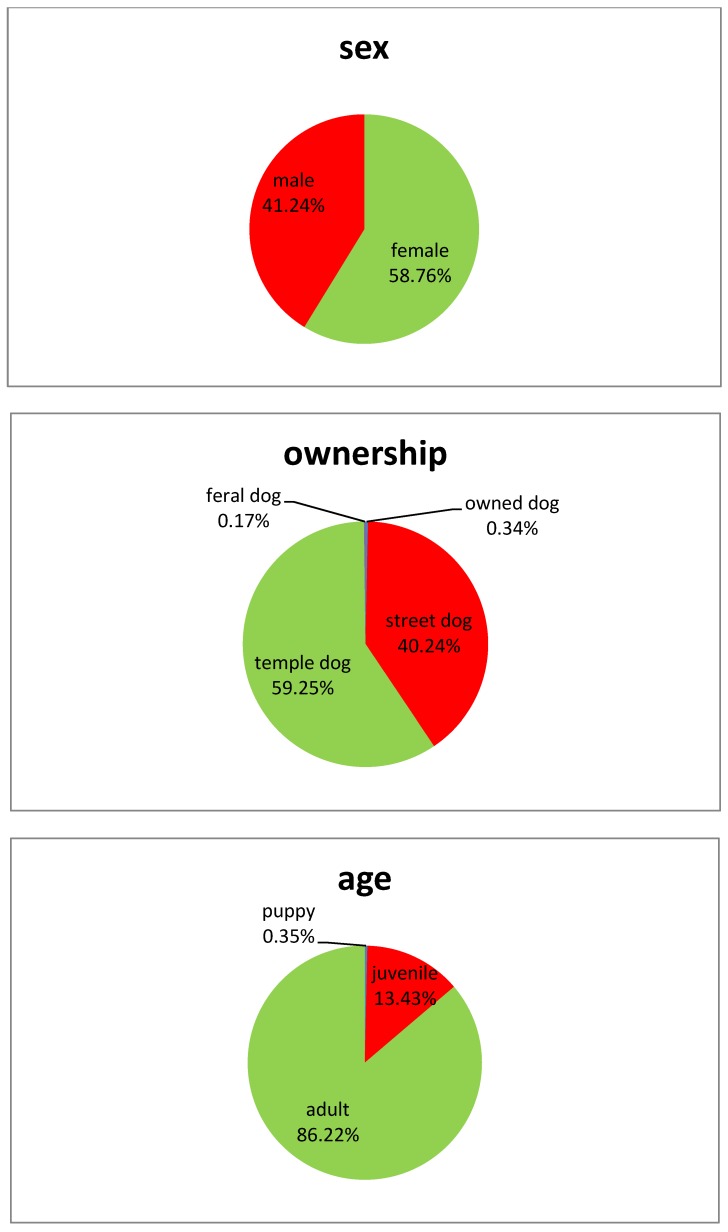
Characteristics of the dog population included in this study.

**Figure 3:**
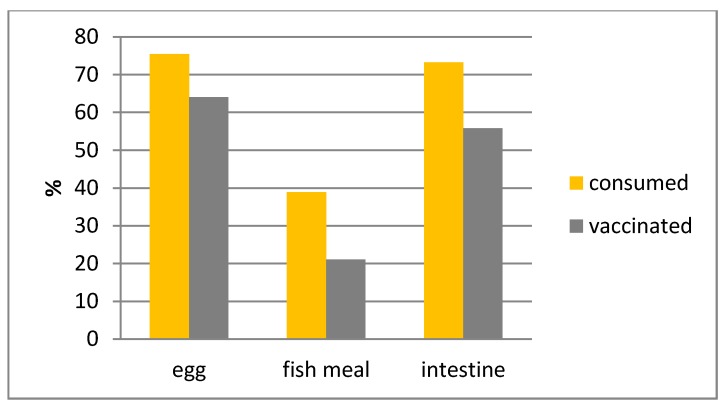
The percentages of dogs consuming the offered bait type and were subsequently considered vaccinated (release of contents of vaccine capsule in oral cavity).

Of all 620 baiting attempts recorded, 604 data sets were used for analysis. Hence, 16 attempts were removed for different reasons; e.g., conflicting data was entered, bait attempts were interrupted because dogs offered a bait were chased away, dogs buried the baits, or brought it to their puppies. It was not always possible to fill out the complete form. If no data was available for a specific parameter investigated (marked as “unknown”), the data set was not included in the analysis for this particular parameter. Ownership, gender, age classification, and body size for the target dog population were documented and summarized (Figure 2). Overall bait acceptance (showing “direct oral contact”) and consumption was 90.85% and 69.60%, respectively (Table 2). The fish meal bait was significantly more often refused than the other two baits (Chi^2^ = 17, df = 2, *p* < 0.001). There was no significant difference between the three bait types if the capsule was swallowed or discarded (Chi^2^ = 0.33, df = 2, *p* = 0.85). However, capsules in fish meal baits (74.70%) were less often perforated than in the egg baits (93.19%) and intestine baits (90.15%) (Chi^2^ = 17, df = 2, *p* < 0.001). Overall, there was a highly significant effect of bait type on “efficacy” (Chi^2^ = 23, df = 2, *p* < 0.001). The fish meal bait had a significantly lower “efficacy” rate than the other two baits. The content of the capsule was considered successfully released in the oral cavity of 54.85% of the dogs that consumed the fishmeal bait. For the egg and intestine bait, 84.50% and 76.61% of the dogs were considered successfully “vaccinated”, respectively. If the number of dogs that did not accept the bait offered is also included, the proportion of dogs considered “vaccinated” is further reduced; egg bait—64.00% [95% CI: 56.41–71.04], fish meal bait—21.05% [95% CI: 15.49–27.55] and intestine bait—55.81% [95% CI: 48.06–63.27] (Figure 3). Hence, not only was the acceptance of the fish meal bait much lower than the other two bait types tested but also the efficiency in delivering the vaccine in the oral cavity was also lower. 

Temple dogs accepted the baits significantly more often than street dogs (Chi^2^ = 11, df = 1, *p* < 0.001). The intestine bait was better accepted by temple dogs than by street dogs (Chi^2^ = 9.3, df = 1, *p* = 0.002). The higher bait acceptance of temple dogs compared to street dogs for the egg bait (*p* = 0.17) and fish bait (*p* = 0.05) were not significant. As only one feral and two owned dogs were included in the study, these animals have not been incorporated in this statistical analysis (Table 3).

Female dogs accepted baits more often than male dogs (Chi^2^ = 5.8, df = 1, *p* = 0.016). As there were no significant differences between the two sexes for the individual bait type (Chi^2^-test; egg: *p* = 0.36, fish: *p* = 0.08 and intestine: *p* = 0.07) and the 95% CI of the overall acceptance for both sexes slightly overlapped (female: 68.70–78.85%; male: 57.21–70.62%), the observed differences between the sexes should not be overrated (Table 4). Both body-size (small and medium) and age (juvenile and adult) had a significant effect on bait acceptance. Small dogs accepted baits significantly more often than medium-sized dogs (Chi^2^ = 12, df = 1, *p* < 0.001) (Table 5). As well, juveniles accepted the baits significantly more often than adults (Chi² = 8.3, df = 1, *p* = 0.004) (Table 6). Juveniles preferred intestine (93.3%) and egg baits (80.77%) over fish meal (72.70%) but the difference was less pronounced compared to adults. The observation that small dogs showed higher bait acceptance can partially be explained by the fact that juvenile dogs were significantly more often small-sized when compared to adult dogs (Chi^2^ = 170, df = 1, *p* < 0.001). 

The percentages “consumed” do not correspond with the percentages listed in Table 2. In the latter the percentages were based on baits consumed irrespective of whether an assessment of the vaccination attempt was known. In Figure 3 the percentage “consumed” is based on the number of observations where both factors were known.

Most baits were offered in the morning and late afternoon. However, no significant effect in bait acceptance and consumption was observed for the time of day the baits were offered to the dogs (acceptance—Chi^2^ = 5.4, df = 4, *p* = 0.25, consumption—Chi^2^ = 11, df = 6, *p* = 0.08) (Table 7). 

**Figure 4:**
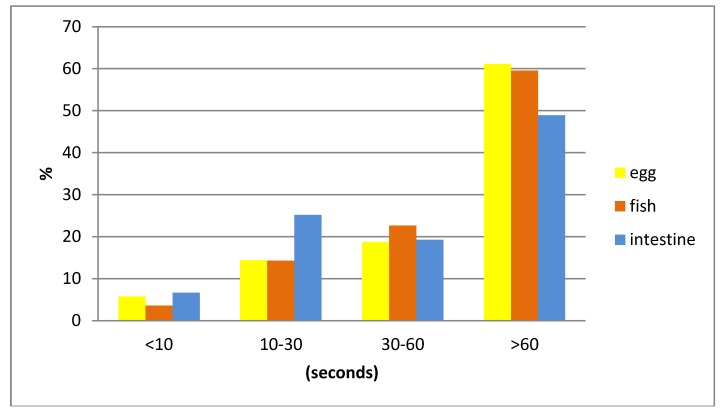
Bait handling time (seconds) for the three different baits.

Generally, single dogs took longer to consume the bait than dogs who were offered a bait when together with other dogs (Table 8). When baits were accepted by the dogs, 83.06% of the animals consumed the whole bait. The intestine bait was most often completely consumed and the fish meal bait most often only partially consumed (Chi^2^ = 15, df = 2, *p* < 0.001) (Table 9). No significant difference in bait handling time between the three different bait types was observed (Chi^2^ = 8.8, df = 6, *p* = 0.19) (Figure 4).

## 4. Discussion

A well-accepted bait by the local dog population is an important pre-condition of the successful implementation of ORV of dogs against rabies. Bait acceptance studies have been conducted in many different countries [16,17,18,19,20,21,22]. Unfortunately, most of the time different baits and study designs were used, confounding a direct comparison. The baits tested and study protocol applied in this study were identical to the ones in a study recently performed in the Navajo Nation, USA, and can therefore be compared directly, except for the small adaptation with the intestine bait. The overall bait consumption and estimated vaccination rate was lower in this study than in the Navajo Nation [15]. Most likely this is a direct result of the exceptional situation in Thailand where most street and temple dogs receive food from care-takers on a daily basis and malnourished dogs are extremely rare. This was also reflected in the prolonged duration of bait handling, especially for the intestine bait, when comparing both studies. In countries where the target population of free-roaming dogs are most of the time undernourished, dogs tend to devour the baits without much chewing, resulting not only in a higher acceptance rate and short bait handling time but also in a much higher swallowing rate of the vaccine capsule. In Thailand, only six dogs (1.72%) swallowed the PVC-capsule. However, 42.86% of all dogs swallowed the same capsule during the field study in the Navajo Nation [15]. Swallowing the PVC-capsule should be avoided since it can cause problems like gastric intolerance in the dogs [23]. However, such adverse events were not reported with the same PVC-capsule during the study in the Navajo Nation and another field trial in Haiti [15,24]. Other factors, such as shape, texture, and size of the bait and capsule can also influence bait acceptance and bait handling, [15,16]. 

Bait acceptance in Thailand was also negatively influenced by the fact that most of the dogs encountered were roaming in groups. Under these circumstances, it is difficult to make sure that every individual animal in the group has access to a bait offered since the dogs compete intensively for the baits. On many occasions, dogs offered a bait were chased away or interrupted by other dogs. Although bait competition among dogs encountered together cannot be circumvented completely, certain bait offering techniques can reduce it to acceptable levels. For example, the first bait offered should always be given to the dominant animal and afterwards baits should be offered immediately to the other dogs but several meters apart from each other. 

No significant difference in bait acceptance was observed between the collagen-baits filled with boiled intestine pieces and the egg-flavored baits in this study. However, the fish meal bait was significantly less often accepted and consumed than the other two baits tested; confirming previous studies with dogs using the same fish meal bait [16,25]. In contrast, 81% of the dogs in Navajo Nation accepted the fish meal bait [15]. Also, a commercially available fish-flavored bait was well accepted (84%) by owned dogs in Sri Lanka [26]. These observations clearly show regional differences in bait acceptance. Baits made from locally available material have generally a higher acceptance rate by dogs than industrial manufactured baits [16,20,21], which is most likely due to the lack of familiarity with the taste, smell, shape, and texture of the bait [16]. The preparation of large quantities of baits made from local food sources is often cumbersome; in this study the initial concept of placing the blister inside a boiled intestine segment was discontinued due to the long preparation time required. Furthermore, locally produced baits are often not favored by field personnel due to difficulties in preparing and handling the baits [21]. Therefore, a well-accepted bait that can be produced in large numbers in a short period of time with standardized composition and quality has distinct advantages. The egg-flavored bait was also well accepted by dogs in Thailand and the Navajo Nation, USA. It was actually more successful in delivering the vaccine in the oral cavity than the intestine bait with collagen casing. However, besides regional differences in bait acceptance by the local dog population, other aspects (such as legal and cultural differences) may hamper the development of a universally deployable manufactured bait for dogs.

The approach used in this study to investigate bait acceptance and handling is based on the so-called hand-out model, one of the three suggested methods of delivering baits to dogs [27]. This method not only reaches the target subpopulation—dogs inaccessible for parenteral vaccination—but also reduces wastage of vaccine baits by recollecting baits not accepted by the dogs and avoiding bait depletion by non-target animals [28]. Another important advantage is that in contrast to the other two suggested methods—the wildlife-model and the distribution of baits to dog owners [27,29]—the hand-out model considerably reduces potential human contact with the vaccine virus. As all oral rabies vaccines are based on live replication-competent human pathogens, direct and indirect contact with these vaccines could lead to adverse events and should therefore be avoided as much as possible [27,30]. By offering the baits directly to the dogs, baits not taken and discarded capsules are recollected, thereby limiting the possibility that humans will come into direct contact with the vaccine [24,31].

The obtained results show that ORV could significantly increase the vaccination coverage of the targeted large free-roaming dog population not accessible for parenteral vaccination in Thailand. Unfortunately, previous serology studies with ORV of dogs in Thailand showed relative low success, with less than 50% showing an immune response [14]. Hence, the next step in evaluating ORV for dogs in Thailand is testing the immunogenicity of a suitable candidate vaccine in local dogs. Thailand has made great progress in reducing dog-mediated human rabies, which was largely achieved by providing relatively expensive rabies post-exposure prophylaxis (PEP) using modern tissue culture vaccines and immunoglobulins [32,33]. The overall costs of PEP have been reduced considerably by introducing vaccination by the intradermal route. Notwithstanding these achievements, large scale administration of PEP can reduce human rabies fatalities but this does not target the problem at its source: the domestic dog. Hence, rabies remains endemic in Thailand. Allocating increasing financial resources to human PEP is not cost-effective and sustainable in contrast to the elimination of dog rabies through mass dog vaccination campaigns [34]. 

## 5. Conclusions

The incorporation of ORV of dogs as part of mass dog vaccination campaign could result in a sufficient number of dogs being vaccinated to interrupt the rabies transmission cycle in its principal reservoir species, reaching the ultimate goal of the elimination of dog-mediated rabies in Thailand. 

## Figures and Tables

**Table 1:** Summary of the three bait types used.

	Material	Size (cm)	Weight (gr)
Bait			
Intestine	Collagen casing filled with pieces of boiled local pork intestine	7–10 cm long	15–25
Fishmeal	vegetable fats + fishmeal	8.5 × 4.0 × 1.2	43
Egg	gelatin + egg powder	8.5 × 4.0 × 1.2	43

**Table 2:** Summary of bait acceptance (“direct oral contact”), consumption and handling, fate of capsule and final assessment if liquid was released in the oral cavity of the dog (“vaccinated”). Except for bait acceptance and consumption, all numbers are based on dogs that (partially) consumed the bait after it was accepted.

Bait-Type	Bait Accepted		Bait Consumed		Capsule Discarded		Capsule Perforated		“Vaccinated”	
	n/N	%	n/N	%	n/N	%	n/N	%	n/N	%
Egg	183/192	95.81	141/179	78.77	133/135	98.52	123/132	93.19	109/129	84.50
Fish	174/206	84.47	86/171	50.29	80/82	97.56	62/83	74.70	40/74	54.85
Intestine	179/192	93.23	137/173	79.19	130/132	98.48	119/132	90.15	95/124	76.61
Total	536/590	90.85	364/523	69.60	343/349	98.28	304/347	87.61	244/327	74.62

**Table 3:** Bait acceptance and the ownership status (street dogs, temple dogs) of the dogs. Only a few owned and feral dogs were included in the study and therefore they were not included in the analysis (n = number of baits accepted, N—number of baits offered, 95% CI—95% confidence interval, n.s.—not significant, sign.—significant).

Bait-Type	Street Dogs					Temple Dogs					Comparison (Chi^2^-Test)
	n	N	%	95% CI		n	N	%	95% CI		
Egg	55	75	73.33	61.86	82.89	82	100	82.00	71.36	87.59	n.s., *p* = 0.17
Fish	26	66	39.39	27.58	52.19	55	100	55.00	44.73	64.97	sign., *p* = 0.05
Intestine	46	67	68.66	56.16	79.44	87	99	87.88	79.78	93.58	sign., *p* = 0.002
Total	127	208	61.06	54.07	67.72	224	299	74.92	69.60	79.73	sign., *p* < 0.001

**Table 4:** Bait acceptance and gender of the dogs (n = number of baits accepted, N—number of baits offered, 95% CI—95% confidence interval, n.s.—not significant, sign.—significant).

Bait-Type	Female					Male					Comparison (Chi²-Test)
	n	N	%	95% CI		n	N	%	95% CI		
Egg	80	99	80.81	71.66	88.03	57	76	75.00	63.74	84.23	n.s., *p* = 0.36
Fish	57	101	56.44	46.21	66.28	28	66	42.42	30.34	55.21	n.s., *p* = 0.08
Intestine	88	104	84.62	76.22	90.94	49	67	73.13	60.90	83.24	n.s., *p* = 0.07
Total	225	304	74.01	68.70	78.85	134	209	64.12	57.21	70.62	sign., *p* = 0.02

**Table 5:** Bait acceptance and body size (small, medium) of the dogs. Only a few large-sized dogs were included in the study and therefore they were not included in the analysis (n = number of baits accepted, N—number of baits offered, 95% CI—95% confidence interval, n.s.—not significant, sign.—significant).

Bait-Type	Small-Sized					Medium-Sized					Comparison (Chi²-Test)
	n	N	%	95% CI		n	N	%	95%CI		
Egg	20	22	90.91	70.84	98.88	113	148	76.35	68.68	82.94	n.s., *p* = 0.12
Fish	17	23	73.91	51.60	98.77	65	140	46.43	37.97	55.05	sign., *p* = 0.01
Intestine	24	25	96.00	76.95	99.90	107	140	76.43	68.52	83.19	sign., *p* = 0.03
Total	61	70	87.14	76.99	93.95	285	428	66.59	61.90	71.05	sign., *p* < 0.001

**Table 6:** Bait acceptance and the age-group (juvenile, adult) of the dogs. Only a few puppies were offered a bait and therefore they were not included in the analysis (n = number of baits accepted, N—number of baits offered, 95% CI—95% confidence interval, n.s.—not significant, sign.—significant).

Bait-Type	Juvenile					Adult					Comparison (Chi²-test)
	n	N	%	95% CI		n	N	%	95% CI		
Egg	21	26	80.77	60.65	93.45	112	141	79.43	71.82	85.77	n.s., *p* = 0.87
Fish	8	11	72.73	39.03	93.98	73	150	48.67	40.43	56.96	n.s., *p* = 0.12
Intestine	28	30	93.33	77.93	99.18	101	131	77.10	68.95	83.98	sign., *p* = 0.04
total	57	67	85.07	74.26	92.60	286	422	67.77	63.08	72.21	sign., *p* = 0.004

**Table 7:** Bait acceptance and—consumption and time point of bait offering (n = number of baits accepted, N—number of baits offered, 95% CI—95% confidence interval).

Time-	Acceptance				Consumption			
Period *	n/N	%	95%CI		n/N	%	95%CI	
6:00 –7:59	12/14	85.71	57.19	98.22	8/13	61.54	31.58	86.14
8:00–9:59	145/156	92.95	87.73	96.43	103/156	66.03	58.02	73.41
10:00–11:59	138/156	88.46	82.38	93.02	103/154	66.88	58.85	74.25
12:00–13:59	30/32	93.75	79.19	99.23	22/30	73.33	54.11	87.72
14:00–15:59	52/61	85.25	73.81	93.03	38/64	59.38	46.37	71.49
16:00–17:59	148/158	93.67	88.67	96.92	89/156	57.05	48.89	64.94
18:00–19:59	12/13	92.31	63.97	99.81	4/13	30.77	9.09	61.43
total	537/590	91.02	88.42	93.2	367/586	62.63	58.57	66.56

*—for statistical analysis, the period 6:00–7:59 and 8:00–9:59 was combined, just as the periods 16:00–17:59 and 18:00–19:59 were also combined.

**Table 8:** The percentage of the different bait handling time periods (seconds) of dogs offered bait when they were alone (single) or together with other dogs.

Bait Handling	Single (n = 88)			Together (n = 265)		
(s)	%	95% CI		%	95% CI	
<10	3.41	0.71	9.64	6.04	3.49	9.62
10–30	9.09	4.01	17.13	21.89	17.06	27.36
30–60	15.91	8.98	25.25	21.13	16.38	26.55
>60	71.59	60.98	80.70	57.11	44.76	57.11

**Table 9:** Proportion of baits completely consumed (n = number of baits consumed completely, N—number of baits consumed, 95% CI—95% confidence interval).

Bait Type	n/N	%	95%CI	
Egg	93/114	81.58	73.23	88.22
Fish	51/72	70.83	58.93	80.96
Intestine	106/115	92.17	85.66	96.36
total	250/301	83.06	78.33	87.12
